# Comparative ethnoentomology of edible stinkbugs in southern Africa and sustainable management considerations

**DOI:** 10.1186/1746-4269-9-20

**Published:** 2013-03-25

**Authors:** Catherine Maria Dzerefos, Ed Tadeusz Fernando Witkowski, Rob Toms

**Affiliations:** 1 University of the Witwatersrand, Restoration and Conservation Biology Research Group, School of Animal, Plant and Environmental Sciences, P. O. Wits, Johannesburg, 2050, South Africa; 2 Ditsong National Museum of Natural History, P. O. Box, 413, Pretoria, 0001, South Africa

**Keywords:** Defence chemical, Edible insects, Entomophagy, Ethnomedicine, Sustainable harvesting, Traditional food

## Abstract

Insects, such as stinkbugs, are able to produce noxious defence chemicals to ward off predators, nevertheless, some ethnic groups have recipes to render them delicious. We provide an example of edible stinkbugs (*Encosternum delegorguei*) used by two locally separate ethnic groups in South Africa, the Vhavenda and Mapulana, with a third group, the Bolobedu using them for commercial purposes. Structured interview schedules and observations with 106 harvesters were conducted to determine differences in use, nomenclature and oral history, methods of collection and preparation as well as perceptions pertaining to availability. The stinkbugs’ foul defence chemical and flight response necessitates nocturnal harvesting when the insect is immobilised by cold. The defence chemical stains the skin and affects vision yet protective gear is not worn. Damage to host trees was recorded when harvesters poached from plantations or private land, whereas, in communal-lands, sustainable methods were preferred. The legitimisation of stinkbug harvesting and introduction of a collection funnel could reduce conflicts with managers of plantations and private land. Two methods to remove the defence chemical for increased palatability were used. Preparation methods differed in whether or not water was used and also whether the head was left intact or removed. Stinkbugs have numerous medicinal uses, in particular as a hangover cure. Awareness and optimal use of beneficial insects, such as stinkbugs, in rural areas could lead to a reconsideration of current environmental management strategies, where harvesters act as habitat stewards and clearing, grazing or burning indigenous vegetation is kept to a minimum.

## Abstract (es)

**Spanish Abstract:**

Superar el temor a la chiche hedionda e implicaciones para una gestión ambiental sostenible

Insectos tales como las chinches hediondas son capaces de producir defensas químicas nocivas para mantener alejados a sus depredadores, a pesar de ello, algunos grupos étnicos han desarrollado recetas para transformarlas en un manjar. Aquí presentamos un ejemplo de chinches comestibles (*Encosternum delegorguei*) utilizadas por dos grupos éticos geográficamente separados que habitan en Sudáfrica, los Vhavenda y los Mapulana, y de un tercer grupo étnico, los Bolobedu, que utilizan estos insectos con fines comerciales. Se llevaron a cabo una serie de observaciones y entrevistas estructuradas con 106 recolectores con la intención de determinar las diferencias en uso, nomenclatura, transmisión oral de conocimientos, métodos de recolección y preparación, así como percepción en relación a la disponibilidad de estos insectos. Debido a las nocivas defensas químicas que posee la chinche hedionda y a su capacidad de vuelo, se hace necesaria la recolección nocturna, cuando el insecto se encuentra inmovilizado por el frío. Sus defensas químicas tiñen la piel y afectan la visión, a pesar de lo cual los recolectores no hacen uso de ningún tipo de protección. Se registraron daños a los árboles hospedadores cuando los recolectores desarrollaban su actividad de manera furtiva en cultivos y terrenos de propiedad privada mientras que en terrenos de propiedad comunal se observó una preferencia por métodos sostenibles de recolección. La regularización de la recolección de las chinches junto con el uso de un embudo para la misma podría reducir los conflictos con los gestores de cultivos y terrenos privados. Se registró el uso de dos métodos distintos para eliminar las defensas químicas de las chinches y aumentar así su palatabilidad. Los métodos de preparación difirieron en el uso o no de agua y en la extirpación o no de la cabeza. Las chinches hediondas tienen muchos usos medicinales, en particular, se utilizan como remedio para la resaca. La concienciación y el uso óptimo de insectos beneficiosos tales como la chinche hedionda en áreas rurales podría permitir un replanteamiento de las actuales estrategias de gestión ambiental en el que los recolectores actuarían como custodios del territorio y actividades como el clareo, pastoreo o quema de la vegetación indígena se reducirían al mínimo.

## Introduction

Ethnoentomology investigates the many, varied interactions between humans and insects [1]. Within the dichotomy of friend or foe, insects can be medicine or a source of poison [2-4], a free food source with protein levels on par or better than meat [5] or a competitor for plant crops [1]. Almost 2000 insect species are consumed globally [6] of which many are regarded a delicacy [7] and could be eaten in preference to fresh meat [5]. In sub-Saharan Africa, 250 edible insect species have been documented in rural areas [8] and can be accessible when areas are drought-stricken and plant crops fail to thrive [9,10]. In Bushbuckridge, South Africa, entomophagy was prevalent in 72% of households (n = 300) (W. Twine, unpublished observations) while towards the north-east in Mametja, 93% of 110 households used 19 insect species such as grasshoppers, termites or flying ants [11]. Data for two villages in Limpopo Province and one in KwaZulu-Natal Province, showed that 68% of 150 households used edible insects [12]. In comparison to entomophagy and medicinal plant research few studies have focussed on medicinal insects particularly in Africa.

**Figure 1:**
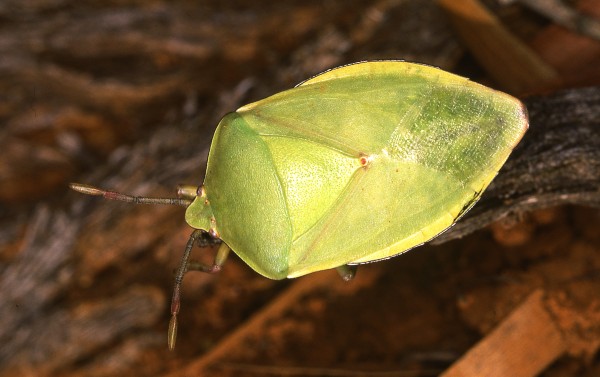
**Dorsal view of the adult stinkbug, *Encosternum delegorguei.***

**Figure 2:**
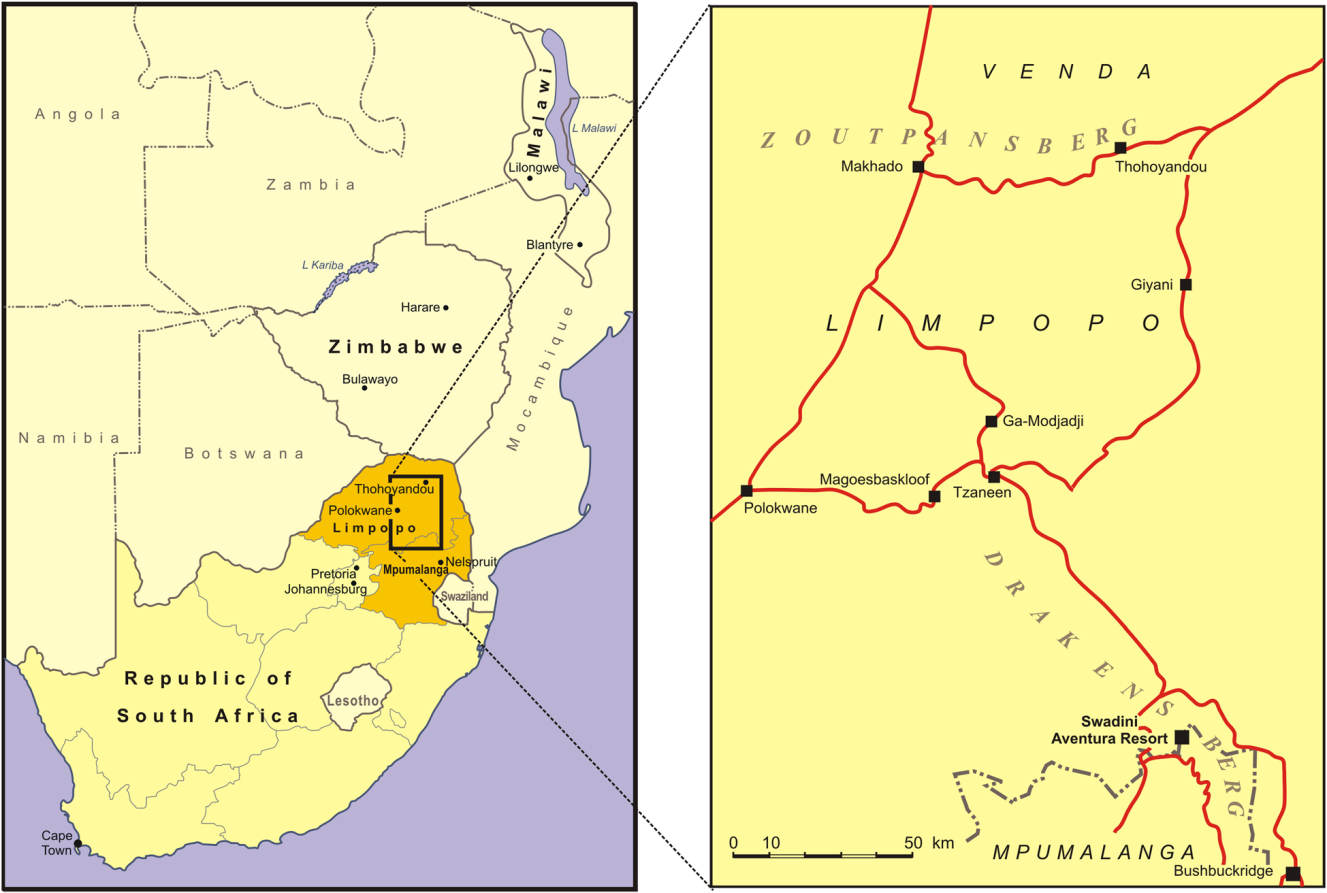
**Edible stinkbug harvesting sites in South Africa.** Edible stinkbug, *Encosternum delegorguei*, harvesting occurs in the foothills of the eastern Zoutpansberg escarpment near Thohoyandou, in the northern Drakensberg at Ga-Modjadji, in Limpopo Province, as well as Bushbuckridge, in Mpumalanga Province, South Africa.

One of the most unexpectedly sought after edible insects in southern Africa is a species of stinkbug, *Encosternum* (=*Haplosterna*) *delegorguei* Spinola (Hemiptera: Tessaratomidae) (Figure 1). It is consumed as a delicacy in south eastern Zimbabwe [13,14] by the Karanga people as well as by two geographically separate ethnic groups in South Africa, the Vhavenda [15,16] and the Mapulana [17] (Figure 2). A Karanga legend recounts the origins of stinkbug use [18]. Nemeso is exiled by his father the chief because he has four eyes. His fortitude is rewarded by the ancestors revealing the secret of rendering stinkbugs palatable.

Stinkbug ‘connoisseurs’ are separated geographically by areas inhabited by people not using stinkbugs. Bolobedu harvesters from Ga-Modjadji are an exception as they have recognised that stinkbugs are a high-value food commodity to the Vhavenda [16] and earn substantial annual income of up to US$ 1105 from sales (Dzerefos CM, Witkowski ETF, Toms R: Commercial and traditional use of the edible stinkbug, *Encosternum delegorguei* (Hem., Tessaratomidae), in press Soc Nat Resour). Stinkbugs are usually abhorred as they squirt a foul defence chemical smelling of rancid almonds [13]. This stains human skin, stings eyes [9] and may cause temporary blindness [17]. Nevertheless, Tessaratomidae and Pentatomidae stinkbugs are collected and eaten raw or cooked in Malawi [19], India [3], Laos [20] Mexico [21] and Papua New Guinea [22]. Analyses of the stinkbugs *E*. *delegorguei*[16], *Atizies taxcoensis* A and *Euchistus sufultus* S [21] indicate good nutritional value. Stinkbugs in southern Africa are ‘harvested’ from trees in woodlands and plantations when the insects aggregate into football-sized clusters, during the winter dry season. This is very convenient for harvesters because at this time of year home grown produce and wild edible plants are scarce [10,23]. The apparently increasing availability of stinkbugs for sale in South Africa has led to a concern that harvesting could be unsustainable [24], and it is therefore crucial to have reliable information on their usage.

The aim of this paper is to provide the first ethnoentomology study on stinkbugs in southern Africa where ethnic differences in local knowledge, nomenclature and use are compared. Specifically, the following key questions have been posed: (1) What does nomenclature and oral history tell us about the use of stinkbugs? (2) Are sustainable methods used to collect stinkbugs? (3) How are stinkbugs prepared to make them palatable? (4) Do users perceive that availability of stinkbugs is changing for some reason? (5) Are stinkbugs available during a drought? (6) Can we suggest measures to assist harvesters and to ensure the sustainable use of stinkbugs and their habitat?

### Study area

Stinkbug aggregation areas around Thohoyandou and Ga-Modjadji in Limpopo Province and Bushbuckridge in Mpumalanga Province, South Africa, were investigated (Figure 2). The sites occur in the transition hill-zone between the escarpment and low-lying plains where winter fires are lit to provide grazing for cattle and goats. The vegetation type is Sour Lowveld Bushveld, of which about 76% is transformed [25] due to natural resource extraction, urban sprawl, subsistence agriculture and commercial crops [26]. A small percentage of the vegetation type is conserved, notably in the Ga-Modjadji Cycad Reserve, the Thate Vondo Forest and the Blyde River Canyon Reserve. Invasive alien plant spread in disturbed areas or along river courses, bush fires, tree felling and overgrazing are negative environmental impacts arising from proximity to growing rural settlements in the study area.

Stinkbug harvesters come from communities with high illiteracy and unemployment [27]. Linkages to urban centres are strong as family members often work there and visit periodically. Although harvesters distinguish themselves ethnically as Bolobedu, Mapulana and Vhavenda other groups such as Sotho and Shangaan are also represented in the villages leading to cultural assimilation. While the convenience of modern shopping is spreading in rural areas, many still use the natural environment for sustenance, medicine and fuel [11,12,15,23,28]. Christianity is widespread but intermingled with ancestral intermediaries to an all-powerful God [29-31]. Even so superstitions and belief in witchcraft is prevalent [32]. Unmonitored access to harvest stinkbugs occurs in plantation forests, communal lands or protected areas. Wildlife is frequently hunted for meat and use in traditional medicine [33]. Water and electricity provision and road infrastructure to rural areas has improved in the last decade but sewage systems remain primitive.

## Materials and methods

From May 2006 to August 2007, 106 stinkbug harvesters were located through word-of-mouth referrals, local print media and revisiting previously described sites [16,17]. Of these, 37 were Mapulana from Bushbuckridge, 29 Bolobedu from Ga-Modjadji, 37 Vhavenda and three Shona from Zimbabwe trading in the Thohoyandou area. A key informant interview schedule was vetted and approved by the University of the Witwatersrand Human Research Ethics Committee (H060524). It was completed with the assistance of field translators. Stinkbug harvesting techniques and post-harvest preparation were documented through experiential observation and semi-structured interviews. Results from interview schedules were presented to harvesters for discussion at a subsequent participatory workshop. Field workers competent in the vernacular languages (Table 1) assisted with translation. Where a household was engaged in stinkbug harvesting, the response of the entire household was entered as the response of one harvester instead of multiple respondents.

**Table 1:** Summary of differences between four ethnic groups utilising stinkbugs in Southern Africa

	**Mapulana (n = 37)**	**Vhavenda (n = 37)**	**Bolobedu (n = 29)**	**Shona (n = 3)**
Origins and description of ethnic group	Mapulana are a sub-group of the northern Sotho	East African and Karanga (Zimbabwe) origins. Of eight Vhavenda sub-groups the Vhatavhatsindi, Vhambedzi and Vhangona eat stinkbugs	Karanga origins, settling first in Venda and finally at Ga-Modjadji. Bolobedu are the people of the Rain Queen and are also known as Balobedu or Lovedu	Karanga
Common vernacular	SePulana	TshiVenda.	Lobedu	Shona
Location	Bushbuckridge Local Municipality, South Africa	Thohoyandou and surrounding villages, Thulamela Local Municipality, South Africa	Ga-Modjadji, Greater Letaba Local Municipality, South Africa	Bikita, Zimbabwe
Colonial era names	Mapulaneng	Venda	Duiwelskloof	Rhodesia
First use of stinkbugs and origin of current use	Recorded in 1944 [17]; pensioners claim to have learnt from grandparents	± 1930; pensioners claim to have learnt from grandparents	1982; claim to have learnt from co-workers at Middlekop tea estate	Recorded in 1905 [13]
Nomenclature	Tsonônô = he farts and is fat	Thungulifha, Dzhovhe, Mbilimedzi from vhilimedza = running after them, Dzama = to die, Fhela = they are scarce, Mbilimedzi khuluvhali = it is hot/very bitter/chillie flavour, Murotho = chemical secretion	Thongolifha, Podile = it is rotten, Morotho = chemical secretion	Harugwa, Harurwa = bitter caterpillar
Collection bag used	A fruit bag with loose weave similar to shade cloth	A maize meal or fruit bag	A maize meal bag	
Storage method	Live stinkbugs kept in fruit bag	Prepared stinkbugs displayed in open containers/spread on bags		
Shelf-life of stinkbugs	After two weeks captivity stinkbugs taste bitter [24]	After six months will taste stale/mouldy		

Interview schedule responses were analysed by comparing (1) preparation methods, (2) perceptions of availability within the last five years, (3) availability in drought years (4) cooperation amongst harvesters and (5) uses. Percentage scores were calculated and where relevant, graphed according to a three-way split across ethnic groups. It should be noted that “no response” data were entered as “don’t know”. Some questions had multiple replies, for example where harvesters identified reasons for stinkbug availability changing and, as a result, over 100% was therefore reflected in these cases.

The Predictive Analytics software (PASW) 18.0 was used for cross-tabulations and Pearson’s Chi-square to determine if harvester ethnicity was associated with perceived availability: (1) in drought years and (2) over the last 5 years. Statistica statistical package V.6. [34] was used for K-Means Cluster analysis, a non-hierarchical cluster method using binary data pertaining to whether or not harvesters (1) consume stinkbugs; (2) sell stinkbugs; (3) remove dead from live stinkbugs; (4) remove the head to prepare living stinkbugs; and, (5) remove the head to prepare dead stinkbugs. Data from the three Zimbabwean harvesters were excluded from statistical procedures due to the small sample.

## Results

### Nomenclature

Vhavenda harvesters use the traditional TshiVenda name “thungulifha” for stinkbugs whereas SePedi speakers use the derivative “thongolifha” (Table 1). Respondents indicated that the Bolobedu started harvesting stinkbugs in Ga-Modjadji communal-lands in 1982 after learning about the commercial value of stinkbugs from Vhavenda co-workers at the Middlekop tea estate in Magoebaskloof. This study identified two Bolobedu households that for the last decade have hosted three to seven Vhavenda harvesters during the stinkbug season. Twenty-two percent of Vhavenda harvesters travel to Ga-Modjadji for collecting. The Vhavenda have a rich vocabulary relating to stinkbug prevalence and taste. An additional movie file documents a Vhavenda woman’s song [see Additional file 1], used after a successful harvesting trip, to inform the community that stinkbugs are available and stating “*we will eat dzhovhe* (stinkbugs) *and mutuko* (sour pap) *on the other side of Mutale* (a river in Venda)”. In Lwamondo, one of the Vhavenda harvesting sites, it is bad luck to say “thungulifha” and the name “mbilimedzi” is used. “Dzama” refers to gravid (having eggs), inedible females that are common from mid-August. The Tshivenda term “fhela” is used to indicate when stinkbugs are scarce. “Mbilimedzi khuluvhali” is used when the stink has not been removed either because the stinkbug was dead on collection or preparation was incorrect. “Mbilimedzi khuluvhali” were said to have a chillie flavour and were known by all ethnic groups to cure hangovers. A Mapulana harvester cautioned that “*if you eat the unprepared one it will kill taste for a month*”. “Podile” is a SePedi generic term for all stinkbugs and is widely used amongst non-eaters.

### Harvesting techniques

Harvesters showed us their hands where short-term exposure to the stinkbugs’ defence chemical stained the skin orange-brown and caused local swelling. They claimed that long-term harvesting (over a decade) caused nails to lift off the nail bed and wart growth. No protective eye-gear was worn, although harvesters said that a direct hit to the eyes burns and affects vision for three days. To protect themselves from the stinkbugs, harvesters usually wore multiple layers of clothing with the neck and sleeves tightly closed. Very few used protective hand-gear such as rubber gloves, woollen mittens or plastic bags as these tended to tear on thorns or slowed-down the collection.

As insects are cold-blooded, the cooler temperatures between dusk and dawn immobilise the stinkbugs. When they are warmed by the sun, stinkbugs fly-away or drop to the ground and fake death or scurry beneath leaf-litter to escape harvesting. Harvesters climb trees or use wooden crooks up to three-metres long to bend branches and access clumps of stinkbugs. Occasionally branches are sawn-off. The end of a branch is placed in a 25-litre bucket and stinkbugs are brushed-off with the free-hand. When the bucket is about 8-cm full the stinkbugs are transferred to a cord-tied bag (Table 1). The bag is shaken before opening so the stinkbugs are disorientated and cannot fly-away. Shaking causes the stinkbugs to release their defence chemical and the energy involved in this process heats up the bag. Bags are secured onto the harvester by a scarf or jacket.

Pines or indigenous trees were felled surreptitiously to access stinkbugs from Komatiland plantations in Venda and Bushbuckridge and a privately owned farm called Ravenshill (237°36^′^02.4″S; 30°16^′^36.5″E) near Ga-Modjadji. Trees next to firebreaks or roads were vulnerable to illegal felling as they fall into a clearing and can be picked clean. The apical points of small pine trees could be damaged when harvesting stinkbugs which results in an inferior tree. In Ga-Modjadji, harvesters said they did not fell trees as the traditional authority had to give permission. Instead harvesters climb trees or hook branches. Some Bolobedu harvesters indicated that the trees should be retained so that stinkbugs would return each year. A Vhavenda harvester confirmed that in Ga-Modjadji, unlike Venda, there is no change in the stinkbug crop as the Bolobedu are not felling trees.

### Availability of stinkbugs

Stinkbugs overwinter in large numbers in clusters which make harvesting worthwhile. Almost half the harvesters (48%) indicated that since 2002 the availability of stinkbugs had decreased whereas 19% perceived an increase (Table 2). There was no association between ethnicity of harvester and the perception of availability over the last five years (χ^2^ = 8.401; *df* = 4; *P* = 0.078). Diverse reasons pertaining to anthropogenic change (plantations, crops, fire and over-exploitation) and weather patterns (strong winds, too much/too little rain) were given for perceived change, although 33% of harvesters could not provide a reason. Most harvesters believed drought had influenced a decrease in stinkbugs and this perception was dependent on harvester ethnicity (χ^2^ = 25.569; *df* = 2; *P* < 0.0001) and cited by 59% Bolobedu, 22% Mapulana and 10% Vhavenda.

**Table 2:** **Harvester’s perceptions on whether availability of *Encosternum delegorguei *** **had increased or decreased from 2002 to 2007**

	**Mapulana (n = 37)**		**Vhavenda (n = 37)**		**Bolobedu (n = 29)**		**Total (n = 103)**	
	**n**	**%**	**n**	**%**	**n**	**%**	**n**	**%**
Increased	4	11	6	16	10	34	20	19
Don’t know	20	54	14	38	0	0	34	33
Decreased	13	35	17	46	19	66	49	48
Top three reasons given for decrease	Don’t know (54%), drought (22%), removal of plantations (8%)		Don’t know (46%), establishment of crops (16%) or plantations (16%)		Drought (59%), fire (21%), no other reason given		Don’t know (33%), drought (27%), fire (7%)	

### Stinkbug preparation

**Figure 3:**
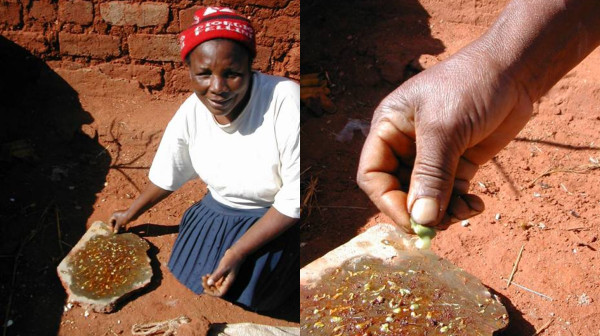
**A Vhavenda woman removes heads and squeezes out the stink glands of edible stinkbugs (*Encosternum delegorguei*) onto a flat-rock.**

**Figure 4:**
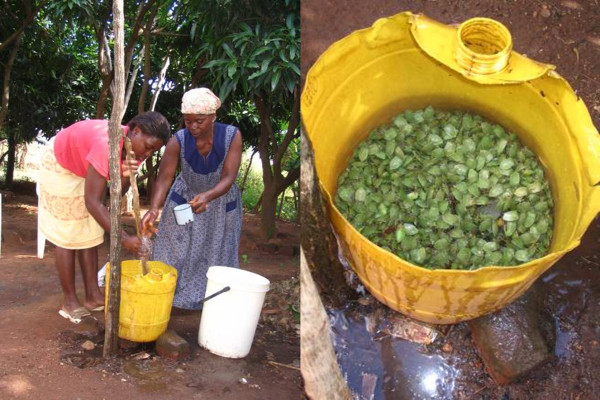
**Bolobedu women kill edible stinkbugs with hot water.** Bolobedu women cause live edible stinkbugs (*Encosternum delegorguei*) to release their defence chemical before dying by pouring hot water over them and stirring with a wooden stick. The contaminated water drains out of the perforated bucket and the air is foul from the released chemical.

Post-harvest sorting of live from dead stinkbugs was done by 89% of harvesters, 10% didn’t sort and 1% occasionally did. The removal of the defence chemical is paramount to stinkbugs being a table delight and two methods of preparation have been documented (Table 3). The Mapulana, and occasionally the Vhavenda (Table 4), used the traditional time-consuming method of removing heads and stink glands (Figure 3) and eating the stinkbugs on the day of preparation. The Vhavenda may also use the modern water-method favoured by the Bolobedu and Zimbabweans (Table 3; Figure 4). The water-method left the head intact and allowed many stinkbugs to be processed at one time for maximum profit and extended shelf-life. Total respondents, using the two methods were not markedly different, with 45% removing and 55% leaving heads intact (Table 4). A Vhavenda source claimed that the traditional waterless preparation method was started by cattle herders when water was unavailable. Storage methods were simple (Table 1) and shelf-life of living and dead stinkbugs was less than six months.

**Table 3:** **Two methods to prepare *Encosternum delegorguei *** **for consumption in South Africa**

**Waterless method (used by Mapulana and Vhavenda)**	**Water-method (used by Bolobedu and Vhavenda)**
**Steps pre-braising:**	
The stinkbug head is held between thumb and forefinger and nicked off on to a flat-rock.	Bagged stinkbugs are shaken vigorously and dropped into a 25-litre bucket with a perforated bottom.
Squeezing releases the thoracic contents. Storage does not occur as they proceed to braising immediately.	
	Hot water is poured over the stinkbugs and they are stirred quickly with a long pole or spoon.
	The stinkbugs release their defence chemical and within five minutes are dead.
	They are rinsed with a bucket of cold-water and transferred to a pot of water heated to about 50°C for eight minutes.
	The water is drained off and the stinkbugs are spread on bags on the floor to air dry.
	Stinkbugs that were dead at the start don’t release their chemical and are identified by black markings on the thorax [24] and bitter taste. Blackened stinkbugs are removed.
	Dried stinkbugs may be stored up to six months.
**Final braising:**	
The detoxified stinkbugs are braised in a frying pan with salt and eaten as a spicy accompaniment to maize meal or alone as a snack.	

**Table 4:** Summary of differences between the post-harvest preparations (% harvesters) in four ethnic groups utilising stinkbugs in Southern Africa

	**Mapulana (n = 37)**	**Vhavenda (n = 37)**	**Bolobedu (n = 29)**	**Zimbabwean (n = 3)**
Remove live head and scent gland	100	24	0	0
Use water on live stinkbugs leaving head intact	0	76	100	100
Remove dead head and scent gland	8	22	0	0
Use water on dead stinkbugs leaving head intact	0	78	0	0

**Figure 5:**
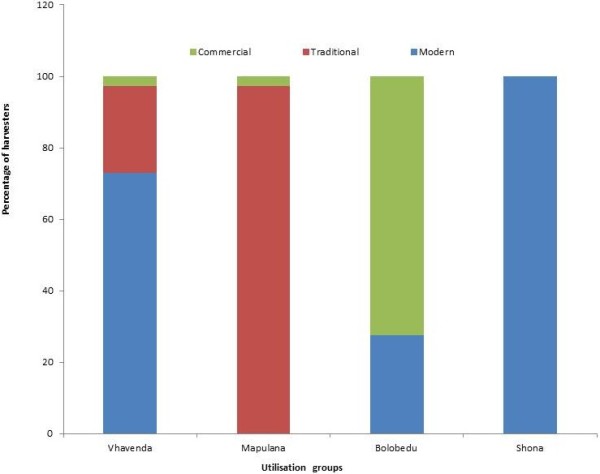
***Encosternum delegorguei *utilisation groups determined by K-means cluster analysis.** Edible stinkbug (*Encosternum delegorguei)* utilisation groups (i.e. modern, traditional or commercial) determined by K-means cluster analysis and based on preparation methods and whether harvesters eat and/or sell stinkbugs, where n = 106 harvesters.

K-means cluster analysis identified three homogenous user groups that correlated with the ethnic groups, and also identified respondents that were using stinkbugs in ways not consistent with the rest of their group (Figure 5). The Vhavhenda displayed more variation in their utilisation patterns (73% behaved ‘modern’, 24% traditional, and 3% commercial), whereas the Mapulana utilisation patterns varied the least and were mostly traditional (97%). Modern users ate stinkbugs and used the quicker water method of preparation which left the head intact. The Mapulana only used the waterless method of preparation and all but one harvester consumed stinkbugs. The Bolobedu dominated the commercial group which tended to not eat stinkbugs. Eight Bolobedu were in the modern group, which was dominated by Vhavenda and included the three Zimbabweans.

Dead stinkbugs were used to cure hangovers, thrown away or prepared and eaten by some Vhavenda (22%) and Mapulana (8%). A variety of traditional medicinal uses were mentioned such as curing headaches and sore throats, controlling diabetes, treating arthritis or skin cancer. The blackish water left from preparing the stinkbugs was deposited in a corner of the yard. The Vhavenda believe that throwing the dirty water on paths or places where people walk will bring bad luck or poison trees.

**Figure 6:**
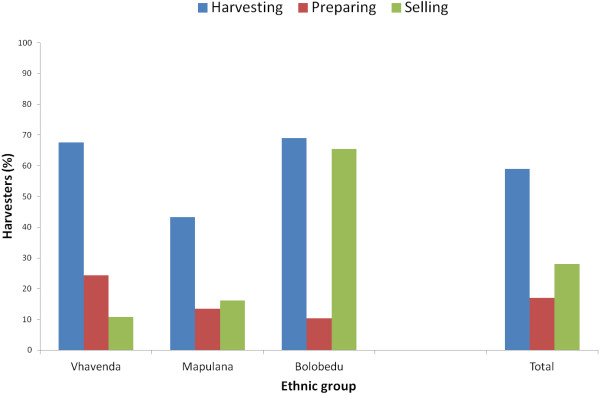
**Cooperation between edible stinkbug harvesters in South Africa.** Cooperation between edible stinkbug harvesters when harvesting, preparing and selling the edible stinkbug, *Encosternum delegorguei,* in three ethnic groups in South Africa, the Vhavenda, Mapulana and Bolobedu, where n = 103 harvesters.

Stinkbug harvesting tended to be matriarchal with 73% Vhavenda, 62% Mapulana and 100% Bolobedu and Zimbabweans being women harvesters. Cooperative harvesting occurred with 59% of harvesters while 28% would sell together and 17% prepared stinkbugs together (Figure 6). Mapulana harvesters were the least likely to cooperate with one another. Sixty-six percent of Bolobedu prefer cooperative selling to sole trading as then one harvester would travel to Thohoyandou to sell while the rest continued harvesting.

## Discussion

“*If you have Thungulifha you can leave the meat!*” This startling statement by a Vhavenda harvester demonstrates the significance of stinkbugs as a food, particularly a source of protein, and suggests that large-scale production as a mini-livestock has merit. In contrast, Sotho and Shangaan people use derogatory names such as stinkbug or “podile”. Such terms do not promote insect conservation which needs to extend beyond the fences of protected areas [35,36] and into private gardens and communal-lands. Stinkbug harvesting represents overcoming a fear-of-nature as it involves eating an organism that is usually abhorred. Fear-of-nature is a common phenomenon in Africa and has inadvertently assisted conservation. Many water bodies [30,37], mountains or forests [31,38] are associated with ancestral spirits [39] or fearsome figures like lions, crocodiles, snakes and mermaids, which keeps these sites revered and pristine [30]. Education [28], Christianity [38], population growth, colonial policies, infrastructure development and entrepreneurship are factors that erode fear-of-nature in rural areas. Without such taboos, land-use practices that are not aligned with sustainable environmental management proliferate [28,30,33].

The rich vocabulary used in relation to harvesting particularly by the Vhavenda (Table 1) indicates that use may be older than living memory. The Mapulana do not know the TshiVenda name for stinkbugs and *vice versa* for the SePulana name, suggesting that the use developed independently in these groups. Vhavenda and Mapulana harvesters tend to be opportunists who temporarily were in financial straits or were working in an area and came across aggregations of stinkbugs. For example, one respondent stopped selling stinkbugs when she began to receive a child support government grant. Others only harvest when stinkbugs aggregate in plantations rather than indigenous bush, which can be overgrown, thorny or harbour snakes. Another obstacle to harvesting is that stinkbugs are easiest to collect between sunset and sunrise [40] when criminals could target lone harvesters. Consequently many women harvest in groups or involve their families. In Bushbuckridge, school boys wanting pocket money during the school vacations have turned to harvesting. In Zimbabwe income from *I. belina* was used by child-harvesters for school-fees and to purchase stationary [7]. Stinkbug harvesting wanes towards August as the population has been thinned and more effort is required to find them. Harvesters perceived drought to have a negative impact on stinkbug availability.

Climbing trees or hooking and pulling down branches were sustainable harvesting methods observed for collecting stinkbugs. Branches may be cut or accidently breakoff and in Zimbabwe this has resulted in trees with heights below three-metres [9,14]. It is of growing concern that in plantations and private land, poachers damage growing points of young pines and fell mature trees to access stinkbugs. It is important that harvesting in these areas is legitimised so that it can be monitored and the use of a collection funnel [19] could be promoted as an alternative to felling trees. Funnels extend to five-metres and allow for efficient collection of large quantities of stinkbugs [19].

Cooking with salt and spreading out, instead of storing in closed bags or bottles, extended shelf-life of stinkbugs but increased loss to rodents. Insects with a high fat content don’t dry out completely [14,24] and are susceptible to mould. In 100 g of dried stinkbugs, substantial amounts of protein (35%), fat (51%), threonine (0.82 mg), valine (1.32 mg) and mineral content (1.2 g) were found [16] even though edible insects with a high fat content lose nutrients following cooking and drying [41]. Alternative methods of preserving such as vacuum-packing, freeze-drying, canning, or pickling could extend the shelf-life, preserve nutrients and allow for marketing beyond national borders.

Identical nomenclature, preparation techniques, the existing market chain and harvesters confirm that the Vhavenda introduced stinkbug use to the Bolobedu in the 1980s but it is possible that the knowledge originates from Zimbabwe (Table 1). Today Bolobedu harvesters will taste dried stinkbugs to evaluate preparation and freshness but don’t relish stinkbugs as a food [19] although grasshoppers and termites are traditional dishes [31]. The Bolobedu capitalise on the presence of stinkbugs in communal-lands and earn a good income (Dzerefos CM, Witkowski ETF, Toms R: Commercial and traditional use of the edible stinkbug, *Encosternum delegorguei* (Hem., Tessaratomidae), in press Soc Nat Resour).

The domestication of stinkbugs and use as a pesticide and medicine should be investigated further by poverty alleviation programmes to add value to the annual stinkbug crop in rural areas. Waste water from stinkbug preparation is currently thrown away in South Africa but in Malawi it was used as a termiticide [19]. Likewise, the defence chemical of the Litchi stinkbug, *Tessaratoma javanica* Thunburg (Heteroptera: Pentatomidae) killed the ants *Camponotus compressus* (F.) and *Monomorium gracillimum* (F. Sm.) and showed antifungal activity [42]. Another potential source of revenue could be unprocessed stinkbugs with defence chemicals intact. Unprocessed stinkbugs were mostly thrown away but 97% of harvesters said they were a hangover cure. Similarly the Malawian edible stinkbug (*Nezara robusta* Dist) was used for hangovers [19]. It is known that some insects produce complex compounds that can be fatal or medicinal, such as terpenoids in blister beetles (*Mylabris* spp.) and melletin in bee venom (*Apis* spp.) [4].

## Conclusion

Stinkbugs were found to be a sought after traditional food amongst the Vhavenda and the Mapulana yet their full potential as a mini-livestock or as medicine or a pesticide has not been fully investigated or marketed. Exploitation can be improved by land managers and harvesters contracted to collect with restrictions to not fell host trees. When a community obtains economic or other benefits from an ecosystem it is likely to be protected from anthropogenic modification [43,44]. In Asia, for example, entomophagy has coincided with decreased pesticide use [45]. In the past, fear-based traditions sufficed for sustainable environmental management but as communities develop, knowledge-based adaptive management [39,40] where the benefits of biodiversity and ecosystems are acknowledged will be needed to prevent environmental degradation and ensure the survival of stinkbugs and associated indigenous plants and animals.

### Consent

Written informed consent was obtained from harvesters for publication of this report and any accompanying images.

## Competing interests

The authors declare that they have no competing interests.

## Authors’ contributions

CD participated in the study design, conducted the fieldwork, analysed and interpreted the data and drafted the manuscript. RT identified the need for the study and coordination thereof. EW also participated in its design and helped to revise the manuscript for important intellectual content. All authors read and approved the final manuscript.

## Authors’ information

Cathy Dzerefos has researched the ecological and socio-economics of rural areas within the Savanna and Grassland Biomes of South Africa. She is currently a provincial manager for the WESSA Eco-Schools programme which provides opportunity to interact with children and teachers in the outdoor classroom and observe and discuss perceptions of nature.

Prof Ed Witkowski has a chair in plant ecology at the University of the Witwatersrand, Johannesburg (WITS), and is the Director of the Restoration and Conservation Biology Research Group. He is also the champion of the WITS Biodiversity Research Thrust. He has published 136 journal papers, and graduated 34 MSc and 14 PhD students to date.

Dr Rob Toms worked at the Transvaal Museum for more than 20 years. He has published more than 70 articles on the origin of insect wings and metamorphosis, cricket communication and indigenous knowledge with special reference to edible insects in South Africa.

## Supplementary Material

Additional file 1 TshiVenda song used after a successful trip to collect stinkbugs. Click here for file
